# Managing Osteopetrosis in the Complex Polytrauma Orthopedic Patient

**DOI:** 10.7759/cureus.21886

**Published:** 2022-02-03

**Authors:** Kenneth Sabacinski, Michael Booth, Michelle Bramer

**Affiliations:** 1 Orthopedic Surgery, West Virginia University, Morgantown, USA; 2 Orthopedics, West Virginia University, Morgantown, USA; 3 Orthopedic Surgery, West Virginia University School of Medicine, Morgantown, USA

**Keywords:** fracture, bone healing, laminoplasty, spine, polytrauma, orthopaedic surgery, osteopetrosis

## Abstract

Osteopetrosis is a genetic illness defined by defective osteoclasts that are incapable of absorbing adequate amounts of bone. This exceedingly rare disorder has been linked to multiple genetic mutations that have a direct impact on osteoclast function. Osteopetrosis causes bones to become brittle with large amounts of cortical bone formation making patients susceptible to pathologic fractures, pancytopenia, and cranial neuropathies among other sequelae. Known as the “marble bone disease,” this condition can range from as severe as causing death in newborn infants to as mild as an incidental finding of increased cortical thickening in a trauma patient. This case demonstrates an incidental finding of osteopetrosis in a trauma patient who suffered from significant injuries as a result of a high-velocity trauma. The patient was the pedestrian in a car vs pedestrian accident and suffered from a central cord syndrome in his cervical spine, a right humerus fracture, a left subtrochanteric femur fracture, a right tibia fracture, and a right fibula fracture. This case report illustrates the complexity of dealing with a polytrauma patient with osteopetrosis and reviews the literature on the approach to fracture fixation in osteopetrotic individuals. This paper will also discuss current medication recommendations and the current standard of care for optimizing patients with osteopetrosis as well as genetic counseling.

## Introduction

Osteopetrosis is an exceedingly rare genetic disease defined by defective osteoclasts that are incapable of absorbing sufficient amounts of bone. As a result, there are large quantities of cortical bone deposition in a disorganized pattern. In effect, this pathologic process creates a bony environment that is more brittle and prone to fracture. This type of bony deposition is why this disease process was given the name osteopetrosis meaning bone made of stone in Greek in 1904 [1].

The first case ever recorded of marble bone disease has been dated back as early as 350 AD [2]. Since its original discovery in 1904 by German Radiologist Albers-Schonberg, studies have shown that there is a strong genetic component to this disease process. Currently, there are four forms of osteopetrosis that are well-known today which include: malignant autosomal recessive, intermediate autosomal recessive, type I autosomal dominant, and type II autosomal dominant [3]. These four phenotypes of osteopetrosis are named after disease severity and inheritance pattern. Among these different subsets of osteopetrosis, specific genetic mutations have been linked to each different phenotype of the disease. Malignant autosomal recessive has been linked to mutations in TC1RG1, CLCN7, OSTM1, PLEKHM1, TNFSF and SNX10, which affect the development and maturation of the osteoclast and are responsible for the severe and rapid onset of this form of the disease [4]. The intermediate autosomal recessive has been linked to a loss of function mutation in the CAII gene responsible for the enzyme carbonic anhydrase [1]. Lastly, the autosomal dominant phenotype develops due to dysfunction of the chloride channel 7 from a mutation in the CLCN7 gene [5]. Each of the mutations has an effect on the bone resorption capabilities of the osteoclast with differing levels of severity and incidence.

Overall, osteopetrosis is an uncommon phenomenon with incidence rates varying depending on the form of the disease. The autosomal recessive form occurs in about one out of every 250,000 births according to epidemiological studies [1]. On the other hand, the autosomal dominant form is relatively more common with a frequency of approximately one in every 20,000 births [1]. In addition to variable rates of disease, the different forms of osteopetrosis have a wide range of clinical findings on initial presentation. The malignant autosomal recessive form typically presents a few months into life and will present with symptoms related to overcrowding of the medullary space including bleeding, abnormal bruising and frequent infections. This form is often fatal without early intervention [6]. In contrast, autosomal dominant osteopetrosis usually presents as an incidental finding in a patient with a pathologic fracture or with early-onset osteoarthritis [7]. All of the phenotypes of this disease can be very challenging to diagnose and even more difficult to effectively treat.

Osteopetrosis can present in multiple ways with significant variability demonstrated by the stark contrast in clinical presentation between the adult and infantile forms. The infantile form can present with features such as macrocephaly, a broad face, frontal bossing, dental abnormalities, short stature, failure to thrive, chronic congestion, cranial nerve abnormalities, hydrocephalus, and tetanic seizures secondary to hyperparathyroidism [8,9]. Most of these issues are secondary to overcrowding of the marrow due to ineffective bone resorption. This may cause extramedullary hematopoiesis which would result in hepatosplenomegaly, pancytopenia, and recurrent infections. On the other hand, the adult form is typically diagnosed by irregularly thick cortices present on radiographs, which may show pathognomic features of osteopetrosis such as a rugger jersey spine, generalized sclerosis and subcristal sclerosis [10]. Although many times osteopetrosis is diagnosed incidentally, the general workup to make the diagnosis of osteopetrosis includes a history and physical exam, bone radiographs, and mutation analysis. The radiographs are typically the definitive method for establishing the diagnosis of osteopetrosis and the mutation analysis is rarely done except for cases of the rarer infantile form and does not usually affect the treatment plan.

Treating osteopetrosis is difficult from both a medical and surgical standpoint. Medically, the only treatment that has shown to alter the course of the disease is bone marrow transplantation for the severe infantile form which poses significant risks. For the adult form, there have been no current medications that have shown to affect the natural course of the disease. Anecdotally, calcitriol supplementation was thought to decrease the severity of disease by increasing remodeling by stimulating osteoclasts, but current studies have shown that the only indication for vitamin D supplementation is for replacement in a deficient individual [11]. Other medications that have been studied include interferon-gamma and prednisone which have significant side effects with long-term administration [10,12]. Surgically, osteopetrosis poses many challenges in the traumatic setting to the orthopedic surgeon. From a mechanical standpoint, the dense cortical bone may complicate any sort of intramedullary fixation device given the significant heat produced by reaming. Thermal necrosis may also arise with any sort of screw fixation which may complicate fracture healing. In addition, the literature shows complications related to a higher rate of failure for load-bearing plate devices, refracture of long bones with plate devices, increased time to union, longer surgical time, and breaking drill bits. All of these factors have to be taken into account when deciding operative versus nonoperative treatment of fractures in patients with osteopetrosis. The following case illustrates the current recommendations for the management of a patient in the acute, polytraumatic setting from both a surgical and medical perspective.

## Case presentation

A 27-year-old male with a past medical history of alcohol abuse presented after being struck by a car while walking across the street. He had immediate pain in his right arm, right leg, left leg, and neck. He also mentions that he was in a motorcycle accident two weeks prior to the current traumatic incident and has had bilateral numbness and tingling in his hands. Since the current accident, his numbness and tingling have migrated to include his elbows along with his hands. He has never had any surgeries, does not take any medications, does not have any allergies, and denies any notable family history of any illnesses or diseases.

Musculoskeletal examination revealed an obvious right humerus deformity with no open wounds but with diffuse paresthesia throughout the right hand and elbow with an inability to extend any fingers or his wrist. The left upper extremity had diffuse paresthesias throughout the upper extremity from the elbow down. He was diffusely weak throughout the left upper extremity. The right lower extremity revealed an obvious lower leg deformity and an open wound to the right buttock. The left lower extremity had an obvious thigh deformity with a shortened and externally rotated leg with no open wounds. On spine exam, he had four beats of clonus bilaterally, decreased sensation throughout bilateral upper extremities, and an inverted brachioradialis reflex bilaterally.

**Figure 1:**
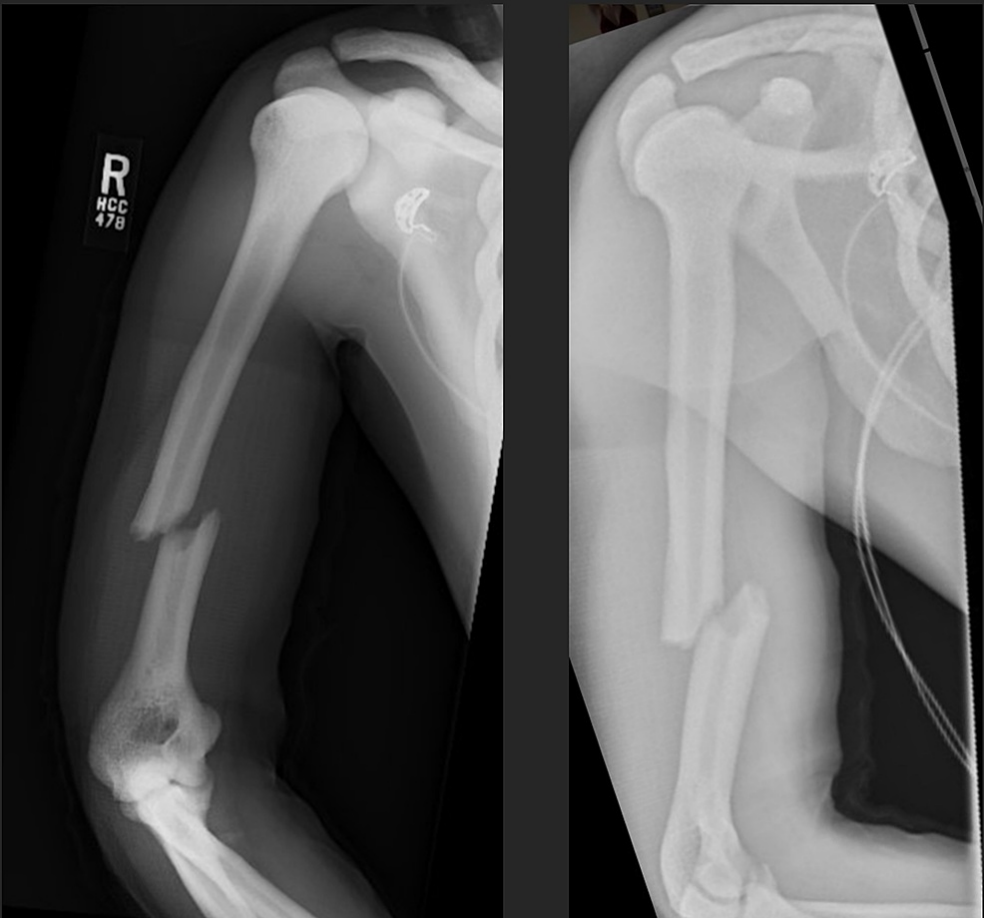
AP (left) and lateral (right) of right humerus fracture. AP - anteroposterior

**Figure 2:**
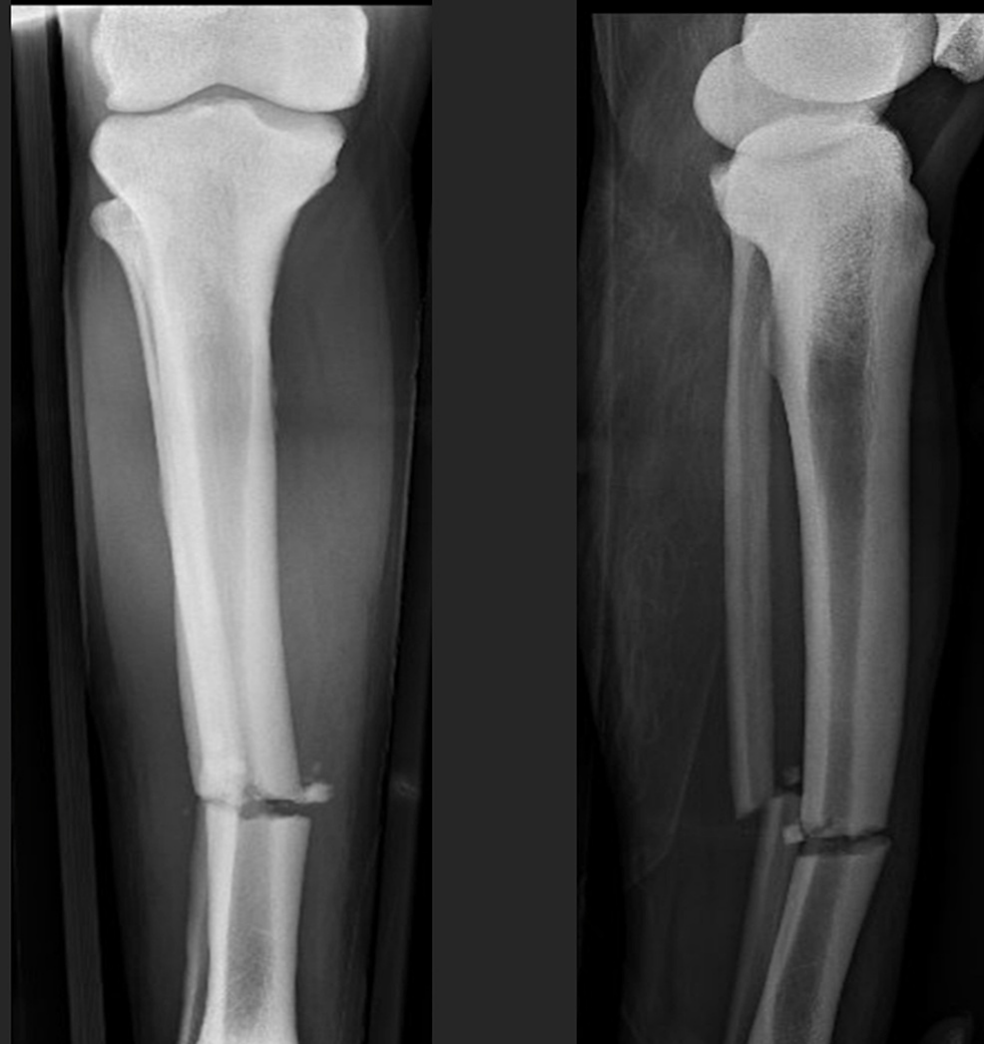
AP (left) and lateral (right) of right tibia fracture. AP - anteroposterior

**Figure 3:**
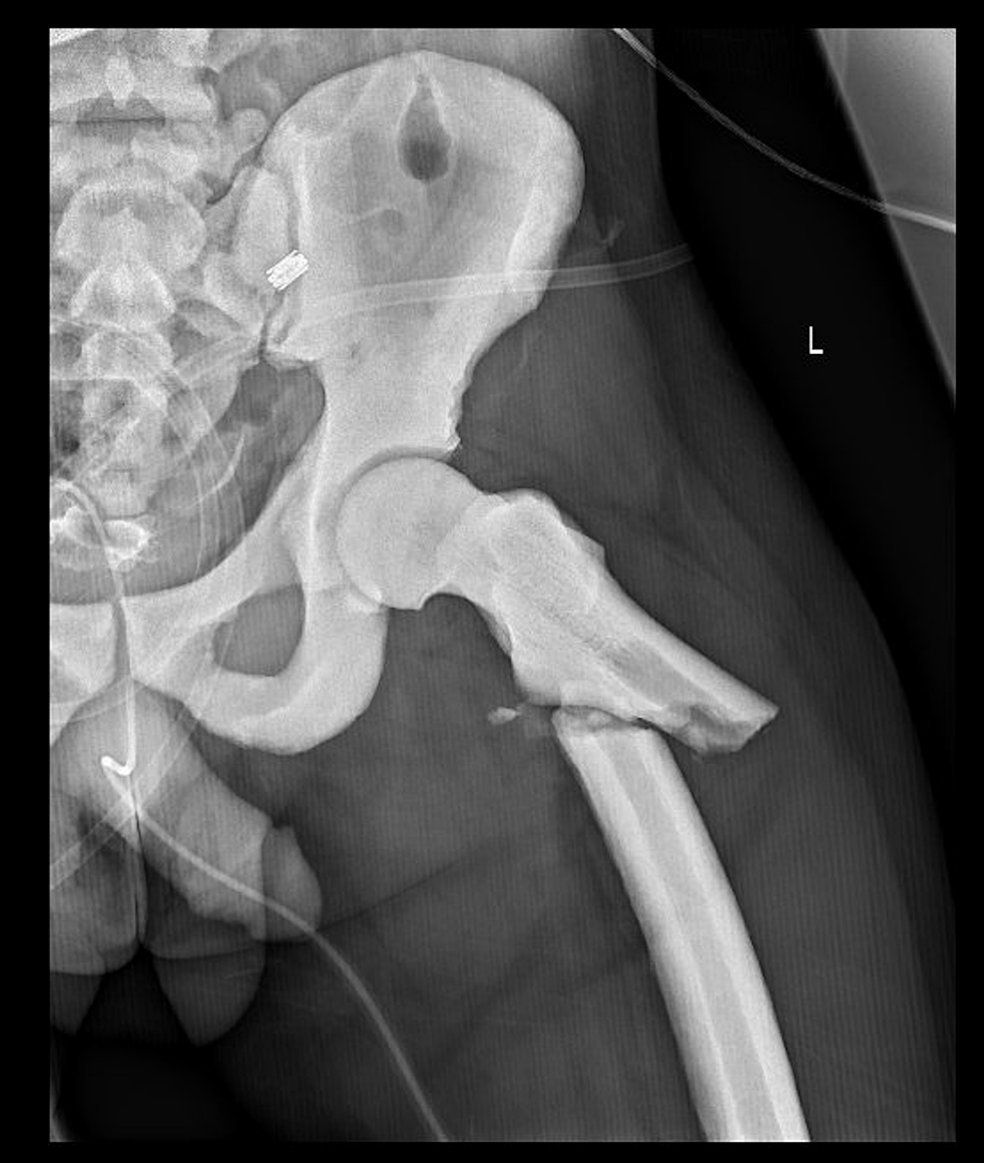
Left femur fracture.

**Figure 4:**
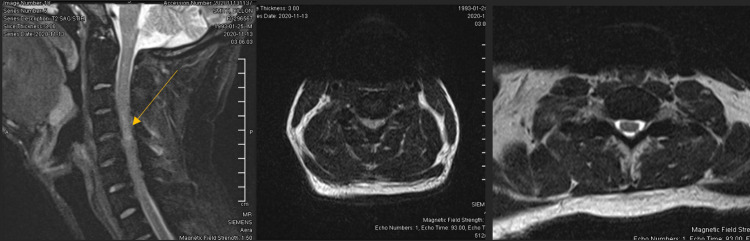
T2 cervical MRI (left) showing cord edema (arrow) at C4-C5 and cord compression on the axial view (center). Note the spinal cord compression and lack of CSF fluid surrounding the cord (center) when compared to the lower cervical spine (right). CSF - Cerebrospinal fluid

Radiographs showed a diffuse sclerotic thickening throughout the extremities and axial skeleton consistent with osteopetrosis. Notable findings from the trauma x-rays were a right distal diaphyseal humerus fracture (Figure 1), a right distal diaphyseal tibial shaft fracture (Figure 2), a left subtrochanteric femur fracture (Figure 3), and a central cord syndrome with signal change on MRI (Figure 4) and a congenitally stenotic canal. Once all of the injuries had been identified, surgical options were discussed with the patient including risks, benefits, expectations, and alternatives to surgery. The patient elected to undergo surgery for all of his injuries including a C3-C7 laminoplasty, open reduction internal fixation (ORIF) of his right humerus, left femur, and right tibia. Given the degree of sclerosis and his small canal diameter for both his femur and tibia were not amenable to intramedullary fixation and underwent plate constructs. The humerus was fixated with a compression plate construct that was contoured to the bone. In terms of sequence, the spine was decompressed and the left subtrochanteric femur fracture was open reduced and fixated with a plate construct and the right tibia and right humerus were fixated at a later date in order to allow for the patient to physiologically recover from the first surgery. Of note, one of the 3.5 mm screws up the left femoral neck had its head break off during insertion. The patient’s MAP was kept above 85 in the setting of central cord syndrome for five days post-operatively. The patient was kept non-weight-bearing on his bilateral lower extremities but was allowed to WBAT to his right upper extremity once ORIF had been completed. The patient was discharged from the hospital after receiving a perioperative ancef and was cleared by physical therapy to be discharged to a rehab facility.

**Figure 5:**
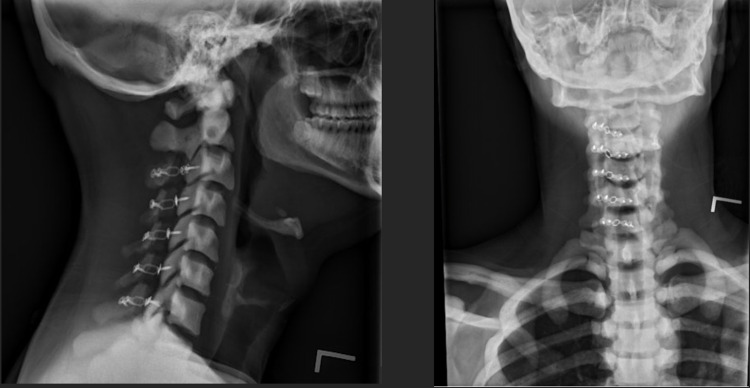
Lateral (left) and AP (right) of c-spine six weeks after laminoplasty. AP - anteroposterior

**Figure 6:**
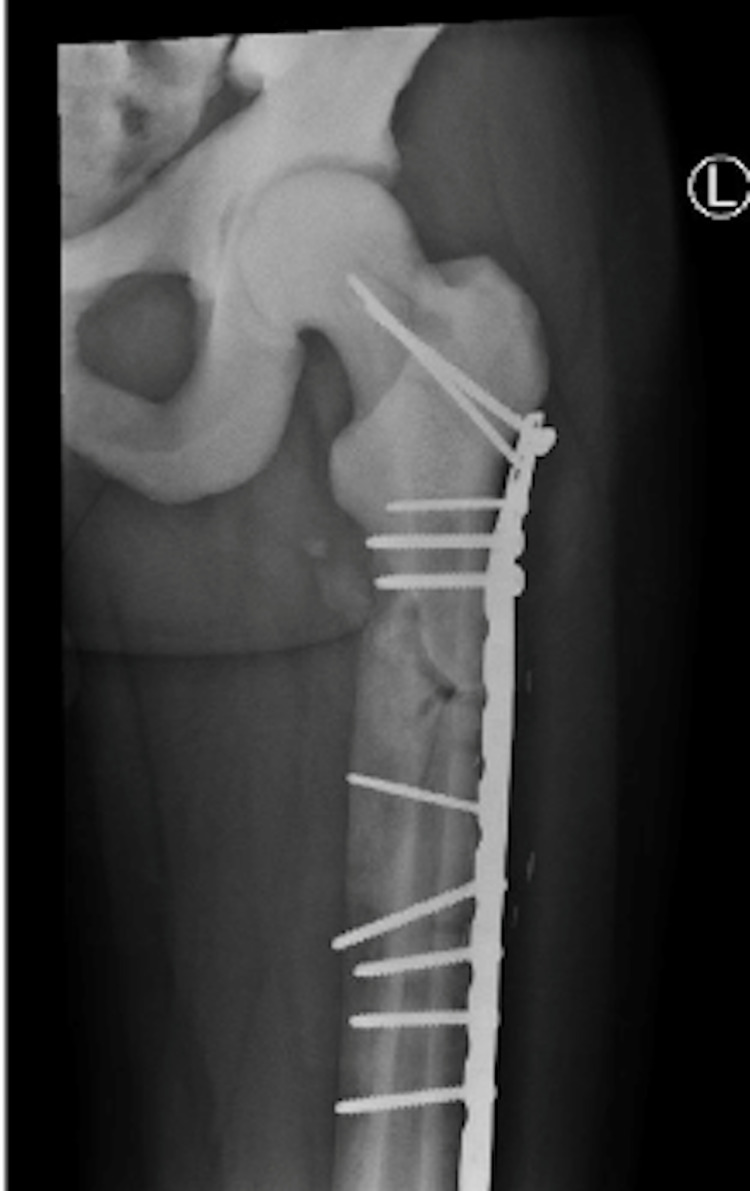
ORIF of left femur six weeks post-operatively. ORIF - open reduction internal fixation

**Figure 7:**
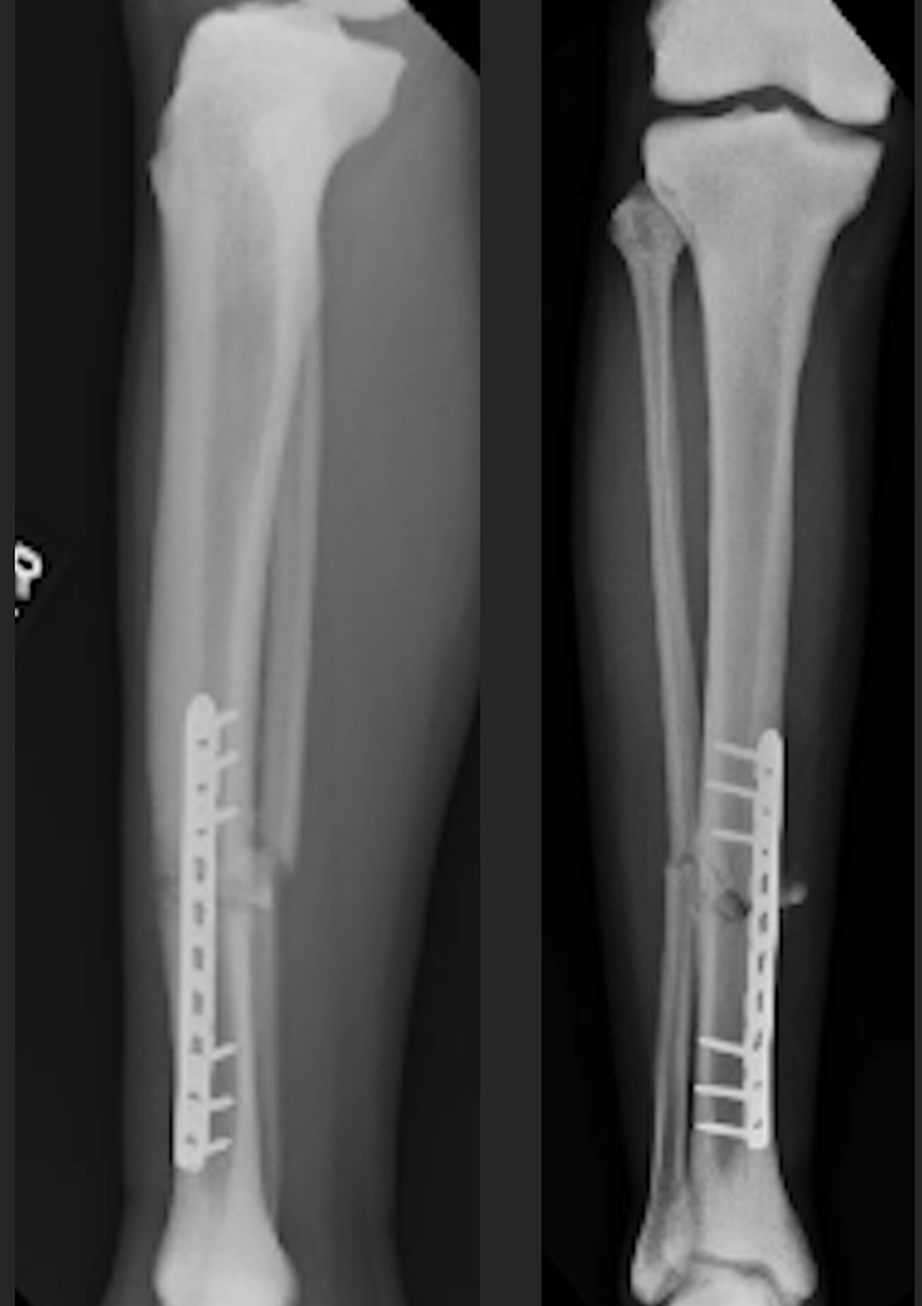
Lateral (left) and AP (right) after ORIF tibia fracture six weeks post-operatively. AP - anteroposterior; ORIF - open reduction internal fixation

**Figure 8:**
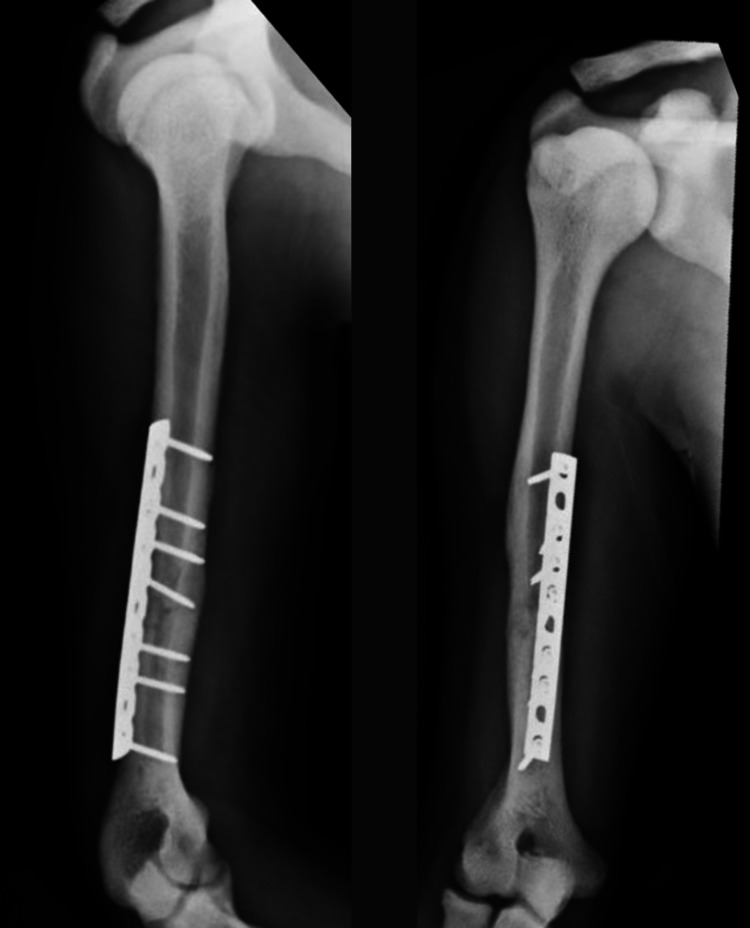
Lateral (left) and AP (right) after ORIF humerus six weeks post-operatively. AP - anteroposterior; ORIF - open reduction internal fixation

**Figure 9:**
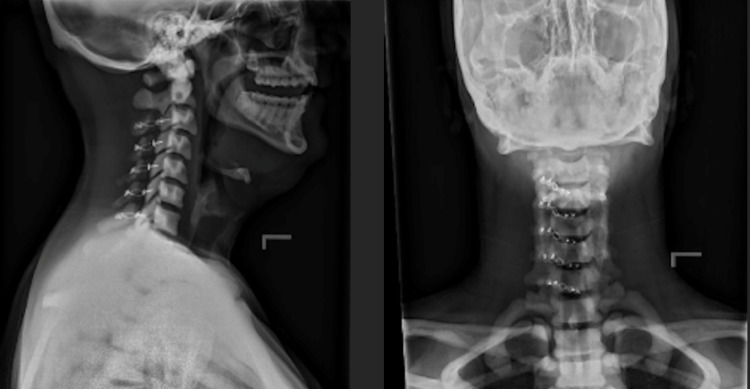
Lateral (left) and AP (right) of cervical laminoplasty three months post-operatively. AP - anteroposterior

**Figure 10:**
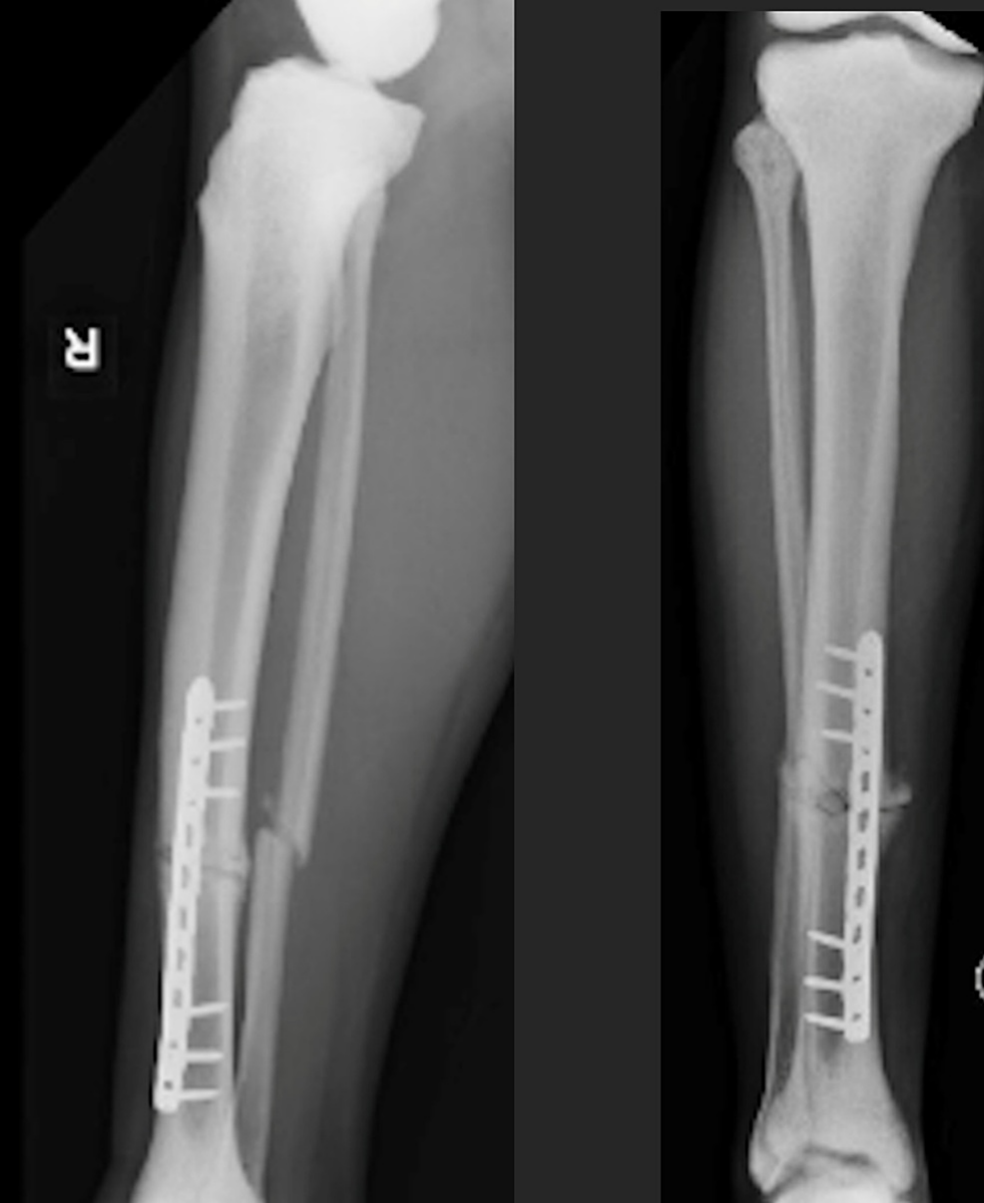
Lateral (left) and AP (right) of ORIF tibia three months post-operatively. AP - anteroposterior; ORIF - open reduction internal fixation

**Figure 11:**
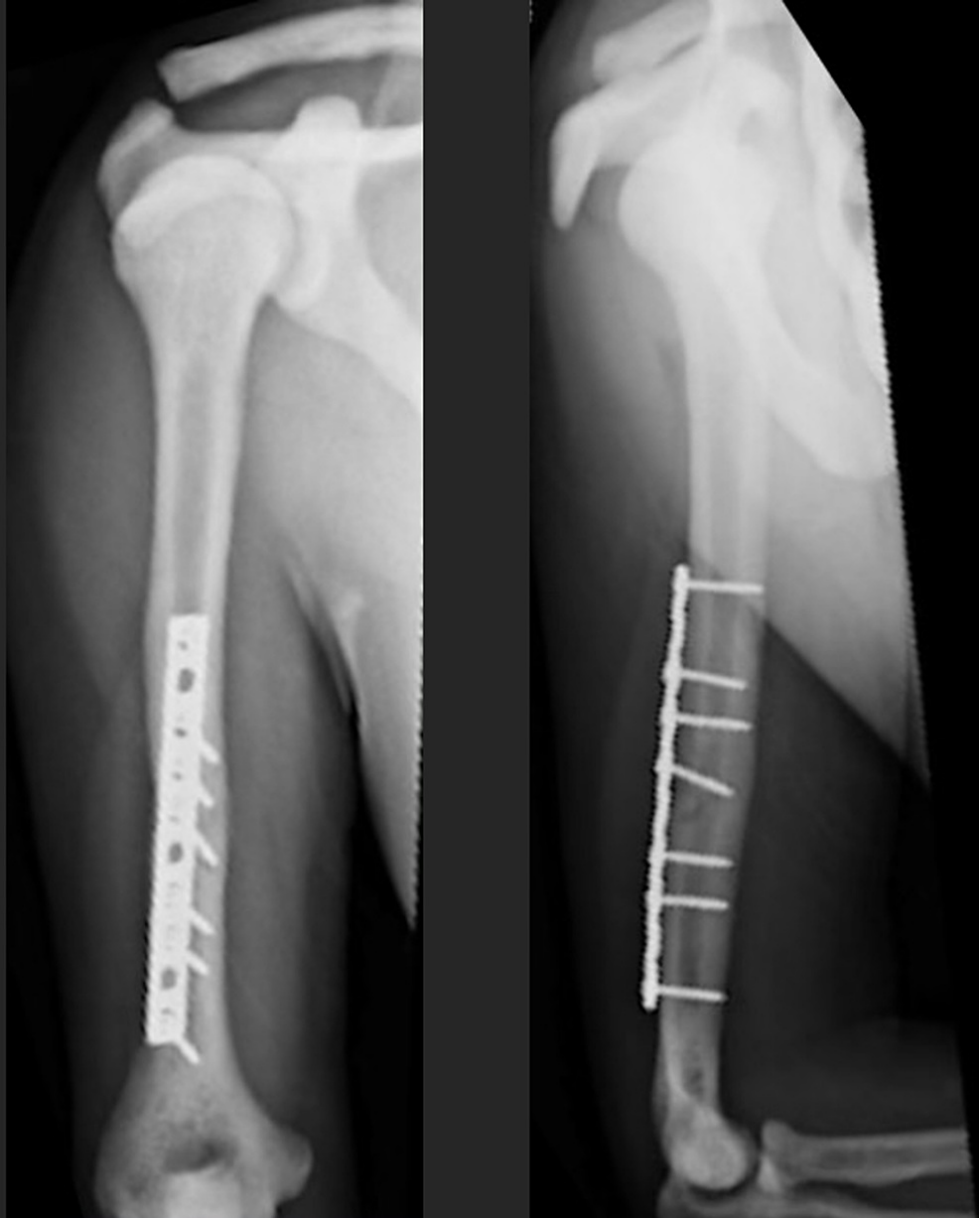
AP (left) and lateral (right) after ORIF humerus three months post-operatively. AP - anteroposterior; ORIF - open reduction internal fixation

**Figure 12:**
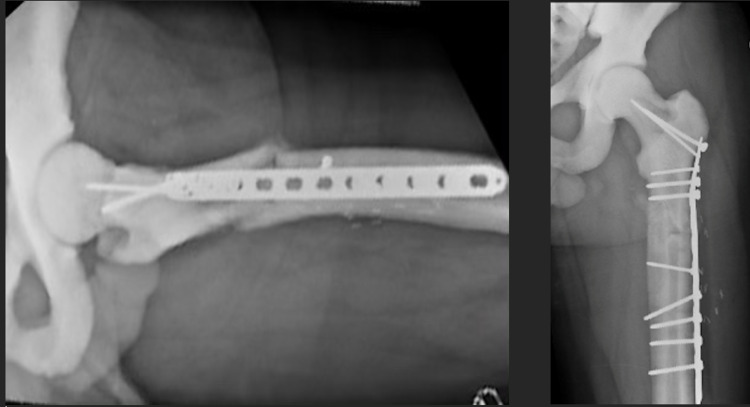
Lateral (left) and AP (right) after ORIF femur three months post-operatively. AP - anteroposterior; ORIF - open reduction internal fixation

At his two-week post-operative visit, he continued to have a right radial nerve palsy consistent with his preoperative examination but otherwise was doing very well at the rehab center. His wounds healed without any complications, and he was advanced to touch-down weight-bearing for his bilateral lower extremities with a full range of motion of all extremities. At his six-week post-operative visit, his right radial nerve palsy was improving and x-rays of his cervical spine (Figure 5) showed stable hardware with no signs of failure. His extremity fractures all appeared to be healing well with no signs of hardware failure or migration (Figures 6-8) and his weight-bearing was advanced to weight-bearing as tolerated. His vitamin D levels were also drawn by the group’s metabolic bone team due to his osteopetrosis and were found to be low (22). At his three-month visit, his vitamin D level was corrected with supplementation and he had improved significantly with his mobility and upper extremity strength. His cervical spine imaging demonstrated stable hardware (Figure 9). His extremity x-rays showed well-healing fractures (Figures 10-12). The patient was lost to follow up after that office visit and returned home in Florida with no issues.

## Discussion

Our case of osteopetrosis is most representative of the adult form. What makes our case unique are the multiple considerations in regards to operative timing, order of fixation, positioning and fixation options. Our decision to decompress the spine and fix the femur first was based on the progressive neurologic deterioration and the morbidity associated with unstable long bone fractures [13].

Our literature review of spinal cord trauma in osteopetrosis yielded limited results but the focus was primarily on the increased propensity for fractures in this patient population. Minor trauma may incite spinal fractures and treatment remains controversial without concrete evidence-based guidelines [14]. The central cord in this case is not surprising given the patient’s mechanism and decreased space available for the cord given the thicker cervical bone. Our case required spinal decompression in the setting of a central cord syndrome which introduced unique surgical challenges. Increased sclerotic bone is anticipated in an osteopetrotic patient. In preparation for surgery, the surgeon must take into account increased thermal injury and possible thermal damage to the spinal cord and have copious irrigation available. In addition to the increased thermal injury intraoperatively, the delayed bone healing associated with osteopetrosis can also influence surgeon preparation and decision making and may push the surgeon towards a decompression procedure such as a laminoplasty as opposed to an arthrodesis.

Osteopetrosis presents several challenges to fracture fixation stemming from the density of cortical bone. Careful reduction maneuvers must be performed to avoid intraoperative fractures and to avoid the increased risk of drill breakage [15,16]. Many operative treatment modalities have been described in the past for subtrochanteric femur fractures including intramedullary nailing, locking compression plate (LCP), dynamic hip screw (DHS), less invasive stabilization system (LISS) plate, dynamic condylar screw (DCS) plating, plaster casting and traction [17-22]. The frequently chosen option for isolated subtrochanteric fracture in these patients is an intramedullary nail because it minimizes blood loss, soft-tissue stripping around the fracture and reduces the bending moment when compared to plate fixation [23]. However, in several case reports, the authors all reported complications ranging from delayed fracture union to infection [24,25]. The issues surrounding the delayed fracture healing in subtrochanteric fractures may be addressed by obtaining a more anatomic reduction and utilizing LCP plating in order to offset the low fracture recovery potential in osteopetrosis [20]. Despite the use of plating for peritrochanteric fracture in osteopetrosis, the reported non-union, delayed union and malunion rate after ORIF is 12%, 40% and 4%, respectively [19].

## Conclusions

Osteopetrosis is a rare osteoclast-mediated disease defined by poor bone resorption due to defective osteoclasts. This case report illustrates a polytrauma patient with central cord syndrome and multiple extremity fractures. After decompressing the spine by performing a laminoplasty, the extremity fractures were addressed using a compressive plating technique that utilized primary bone healing. Intramedullary fixation was considered but not implemented due to the smaller canal size, concern for damaging endosteal blood supply and damaging our intramedullary reamer. When preparing for this type of case, it is imperative to know your implant sizes in order to accommodate the smaller canal. This case supports the hypothesis that osteopetrosis patients heal more successfully utilizing primary bone healing as opposed to conventional fixation techniques. Surgeons may want to consider taking into account the difficulties with secondary bone healing and intramedullary nailing of osteopetrosis patients and consider other forms of fixation with plates and screws to achieve absolute stability and primary bone healing.
